# Pulmonary Carcinoid and the Importance of Correct Radiotracer Selection

**DOI:** 10.31486/toj.20.0155

**Published:** 2021

**Authors:** Nicholas J. Foto, Daniel J. Lee

**Affiliations:** ^1^ Department of Radiology, Ochsner Clinic Foundation, New Orleans, LA; ^2^ The University of Queensland Faculty of Medicine, Ochsner Clinical School, New Orleans, LA

## INTRODUCTION

Positron emission tomography (PET) is a nuclear medicine examination involving the injection of radiotracers that provide a functional assessment of biochemical, physiologic, and pathophysiologic processes. PET radiotracers contain radioactive elements that undergo positron decay and subsequent annihilation with nearby electrons to produce two 511-keV photons traveling in opposite directions at 180 degrees from each other. Modern PET cameras implementing coincidence detection with the help of computer software use this opposite photon trajectory to reconstruct a spatial distribution. Computed tomography (CT) complements the examination to provide anatomic correlation and attenuation correction. Hybrid or fusion PET-CT imaging has become a crucial imaging tool utilized for detection, staging, and treatment follow-up of various benign and malignant conditions.

F18-fludeoxyglucose (F18-FDG) is the most commonly used PET radiotracer. A glucose analog, F18-FDG capitalizes on the known propensity for malignant cells to overutilize glucose by favoring glycolytic metabolism.^1^ Otto Warburg discovered this principle in the 1930s, and it is known as the Warburg effect.^2^ The predisposition for malignant tumors to demonstrate hypermetabolism of glucose provides the basis for F18-FDG PET cancer imaging. While highly sensitive and versatile for imaging a variety of solid and hematologic tumors, F18-FDG has certain limitations, including lack of target specificity because of the ubiquitous utilization of glucose in the body and poor sensitivity for tumors with more indolent behavior and lower utilization of glucose. These F18-FDG limitations have prompted development of several new target-specific radiotracers with more specific mechanisms of localization.^1^ One such PET radiotracer is gallium (Ga)-68 DOTATATE that is commonly used in the imaging of neuroendocrine tumors.

Our case demonstrates how a negative F18-FDG PET-CT examination does not necessarily equate to benignity and how not all malignant lesions are hypermetabolic. We illustrate the importance of understanding the mechanism of localization for PET radiotracers in the evaluation and characterization of unknown lesions.

## CASE DESCRITPION

A 54-year-old male presented to pulmonary medicine with a several-month history of chronic cough. The patient had no other significant medical or surgical history.

## RADIOGRAPHIC APPEARANCE AND TREATMENT

**Figure 1.**
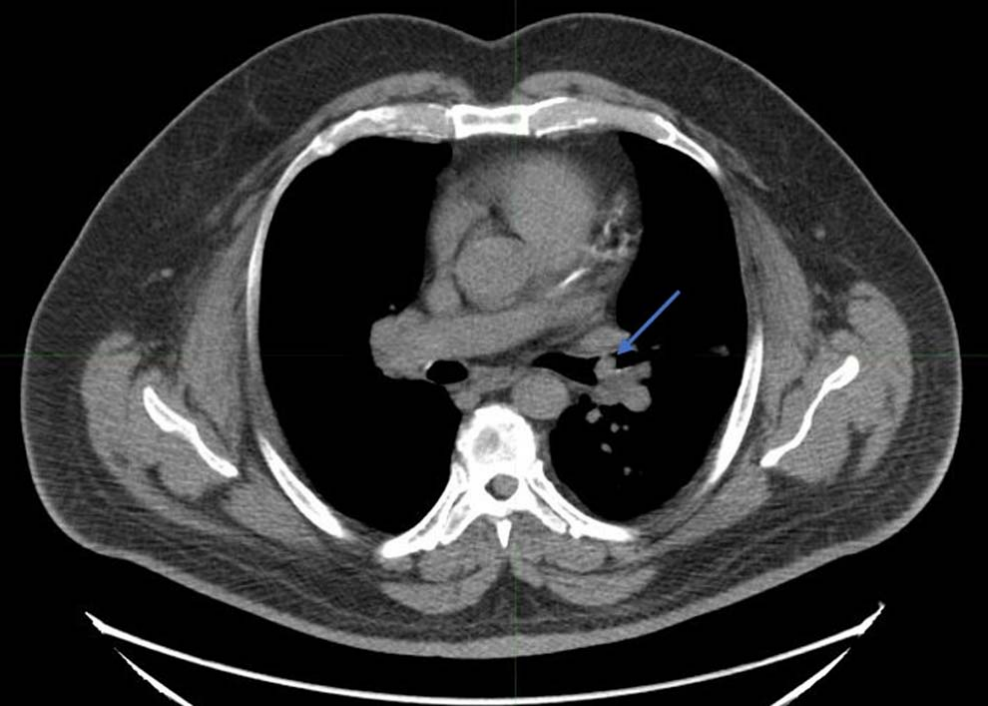
**Noncontrast axial chest computed tomography image demonstrates a 1.3-cm soft-tissue endobronchial lesion within the left upper lobe bronchus, concerning for a primary bronchial tumor or metastasis (arrow).**

**Figure 2.**
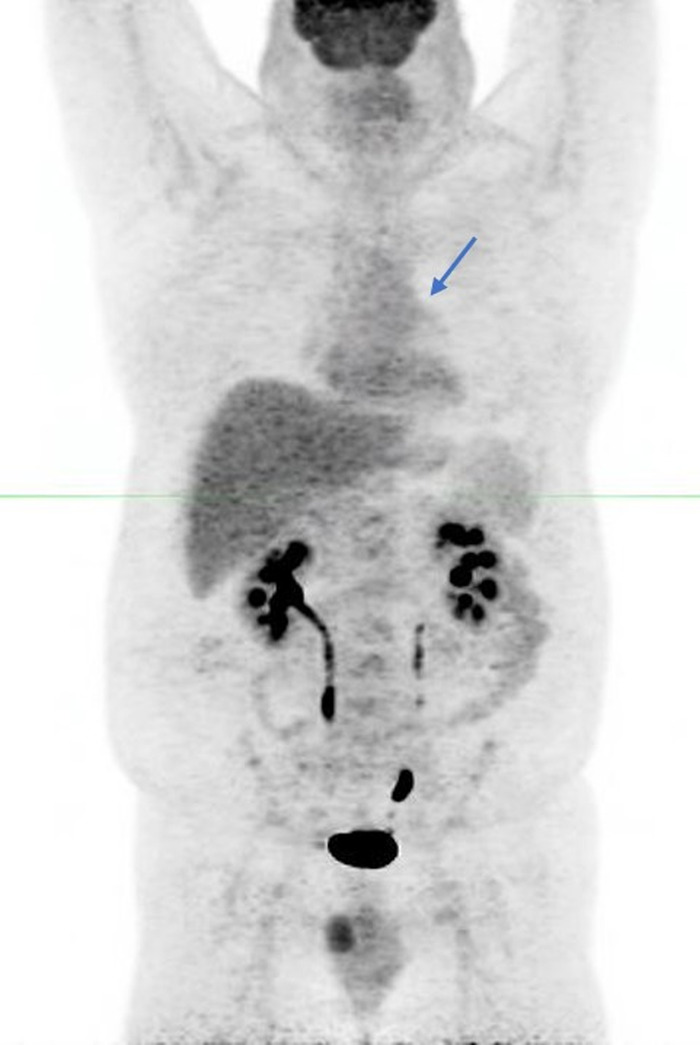
**F18-fludeoxyglucose positron emission tomography maximum intensity projection image demonstrates physiologic radiotracer uptake with no significant activity in the known left upper lobe endobronchial lesion (arrow).**

**Figure 3.**
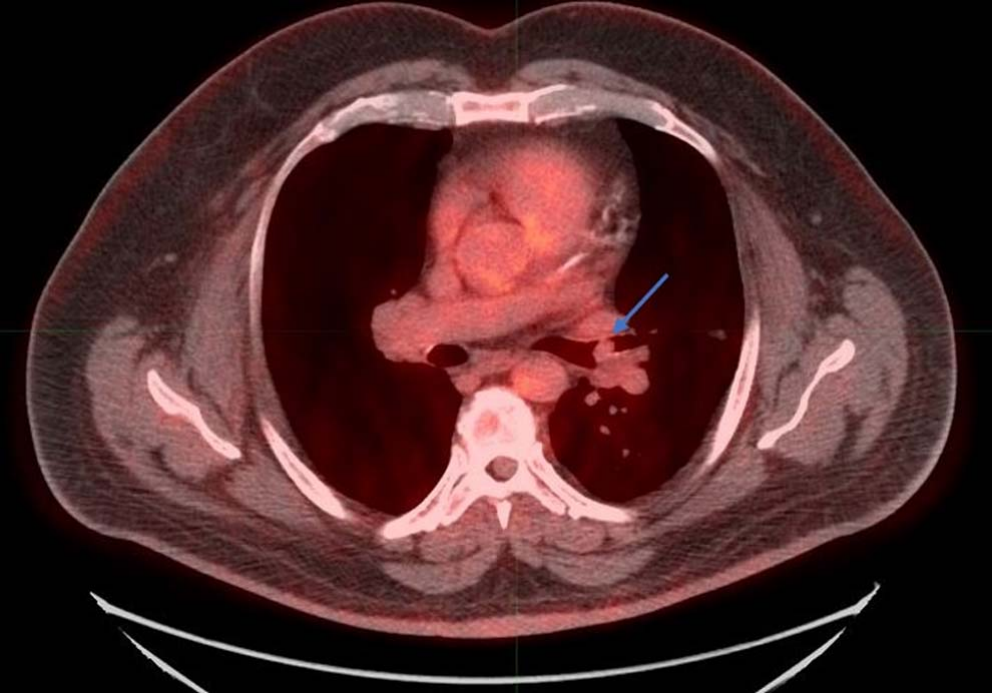
**Fused F18-fludeoxyglucose positron emission tomography-computed tomography axial image shows the left upper lobe endobronchial lesion without significant radiotracer uptake, suggesting a low-grade, well-differentiated tumor (arrow).**

**Figure 4.**
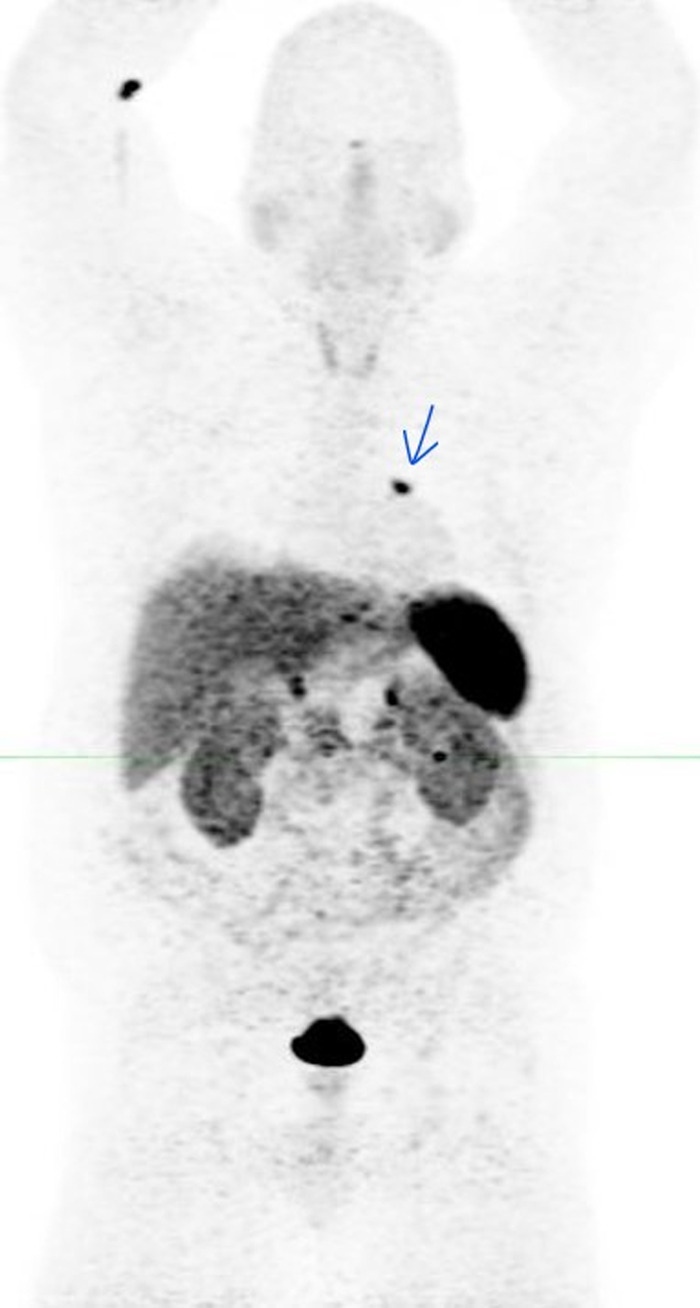
**Gallium-68 DOTATATE maximum intensity projection image demonstrates increased radiotracer uptake in the location of the left upper lobe endobronchial lesion, as well as physiologic distribution in the spleen; liver; bowel; urinary tract; and pituitary, thyroid, and salivary glands. Focal right upper extremity uptake represents the right antecubital fossa injection site (arrow).**

**Figure 5.**
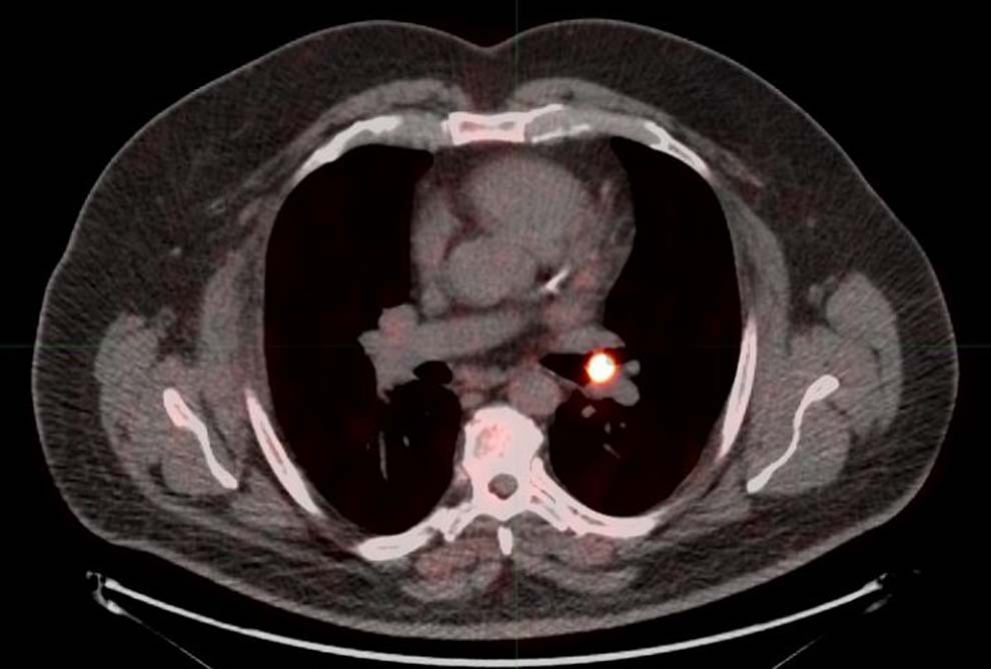
**Fused gallium-68 DOTATATE positron emission tomography-computed tomography axial image demonstrates focal increased somatostatin receptor avidity within the left upper lobe endobronchial mass consistent with a neuroendocrine tumor.**

The patient's symptoms prompted initial evaluation with a noncontrast chest CT that revealed a soft-tissue endobronchial lesion in the left upper lobe bronchus (Figure 1). Incidental finding of left anterior descending coronary artery atherosclerosis was also seen in the image. F18-FDG PET-CT demonstrated no appreciable uptake in the endobronchial lesion, suggesting a low-grade, well-differentiated tumor (Figures 2 and 3). Physiologic uptake was seen in the brain, heart, liver, spleen, gastrointestinal tract, urinary collecting system, and bone marrow. The patient underwent bronchoscopy and biopsy, and pathology was positive for carcinoid tumor. Ga-68 DOTATATE PET-CT demonstrated significant radiotracer uptake in the endobronchial lesion, reflective of the tumor's high somatostatin receptor expression (Figures 4 and 5). Physiologic uptake was seen in the pituitary, thyroid, and salivary glands; spleen; liver; adrenal glands; renal collecting system; and gastrointestinal tract. Incidental uptake in the right antecubital fossa corresponded with the injection site.

Because of the negative F18-FDG PET-CT and lack of distant metastasis shown on the Ga-68 DOTATATE PET-CT, the patient could be considered for surgical excision. Cardiothoracic surgery offered treatment with a left upper lobectomy for tumor resection. The patient underwent surgery, and his chronic cough resolved postoperatively.

## DISCUSSION

Carcinoid tumors are a type of neuroendocrine tumor derived predominantly from enterochromaffin cells. The overall incidence of these tumors is estimated to be approximately 5.25/100,000, with approximately 60% to 70% arising from the gastrointestinal tract.^3^ The second most common site for carcinoid tumors is the tracheobronchial tree, accounting for 25% to 30% of tumors. Given the relatively low incidence of carcinoid tumors, pulmonary carcinoids make up only 1% to 2% of all lung cancers.^4^

Well-differentiated carcinoid tumors are known to have low FDG avidity; therefore, F18-FDG PET-CT has diminished utility in typical carcinoid imaging. FDG avidity increases with tumor grade, so F18-FDG has more efficacy evaluating high-grade, poorly differentiated carcinoid tumors. Neuroendocrine tumor imaging takes advantage of the overexpression of somatostatin receptors on the tumor surface membrane. Carcinoid tumors may express 5 subtypes of somatostatin receptors and are known to express somatostatin receptor 2 most commonly.^5^ Ga-68 DOTATATE contains a synthetic somatostatin analog that binds to somatostatin receptor 2 with high affinity. This radiotracer has shown significantly better accuracy, image quality, and lower radiation dose than indium-111 pentetreotide (octreotide) for neuroendocrine imaging.^6^ The sensitivity and specificity of Ga-68 DOTATATE for detection of neuroendocrine tumors are approximately 80% to 95%.^6^ Ga-68 DOTATATE should be considered when imaging all neuroendocrine tumors such as carcinoid, pancreatic islet cell, medullary thyroid cancer, pheochromocytoma of the adrenal gland, and extra-adrenal paraganglioma.

Fluorine-18 sodium fluoride (F18-NaF) is a bone-specific PET radiotracer that can be used for evaluating osseus metastasis, osteomyelitis, and fracture. This PET radiotracer distributes through the skeleton primarily based on regional perfusion and bone turnover. The specific mechanism of F18-NaF uptake onto the bone surface is known as adsorption, or chemisorption, and leads to tracer integration in the structure of hydroxyapatite.^6^ Technetium (Tc)-labeled diphosphonates are widely used for the detection and evaluation of osteoblastic metastasis because of their availability and relatively low cost; however, F18-NaF PET offers advantages in terms of image quality and lesion detection. With respect to evaluating osteoblastic metastatic disease, F18-NaF offers higher sensitivity (99%) and specificity (97%) compared to Tc-99m methylene diphosphonate (MDP) planar bone imaging alone or with single photon emission CT (SPECT) sensitivity (70% MDP planar and 92% SPECT) and specificity (57% MDP planar and 82% SPECT).^6^

F-18 fluciclovine (Axumin) is a PET radiotracer used to detect prostate cancer recurrence and metastatic disease. This tracer is a radiolabeled amino acid analog that localizes based on the amino acid upregulation known to take place in prostate cancer cells. F-18 fluciclovine has shown sensitivity and specificity for prostate or prostatectomy bed recurrence at 90% and 40%, respectively. In the evaluation of extraprostatic recurrence, sensitivity and specificity are 55% and 97%, respectively.^7^

## CONCLUSION

This case demonstrates the importance of understanding the mechanism of localization for nuclear medicine radiotracers. F18-FDG was the first widely used PET radiotracer for functional tumor imaging and is still the most commonly used radiotracer in the management of cancer patients because of the increased glucose metabolism in most tumors and the increased uptake of F18-FDG seen with many malignancies. However, not all malignant tumors demonstrate these same characteristics, so radiotracers that use different mechanisms of localization are necessary. Having a current working knowledge of the different radiotracers available for PET-CT and understanding that a negative F18-FDG PET-CT does not necessarily exclude malignancy are critical for clinicians. Depending on the biology of a tumor, other PET radiotracers may better identify and show the extent of disease than F18-FDG.

## Notes

This article meets the Accreditation Council for Graduate Medical Education and the American Board of Medical Specialties Maintenance of Certification competencies for Patient Care and Medical Knowledge.

## References

[1] Chen K, Chen X. Positron emission tomography imaging of cancer biology: current status and future prospects. Semin Oncol. 2011;38(1):70-86. doi: 10.1053/j.seminoncol.2010.11.005 21362517 PMC3060704

[2] Miles KA, Williams RE. Warburg revisited: imaging tumour blood flow and metabolism. Cancer Imaging. 2008;8(1):81-86. doi: 10.1102/1470-7330.2008.0011 18390391 PMC2324371

[3] Ganeshan D, Bhosale P, Yang T, Kundra V. Imaging features of carcinoid tumors of the gastrointestinal tract. AJR Am J Roentgenol. 2013;201(4):773-786. doi: 10.2214/AJR.12.9758 24059366

[4] Bertino EM, Confer PD, Colonna JE, Ross P, Otterson GA. Pulmonary neuroendocrine/carcinoid tumors: a review article. Cancer. 2009;115(19):4434-4441. doi: 10.1002/cncr.24498 19562772

[5] Sanli Y, Garg I, Kandathil A, Neuroendocrine tumor diagnosis and management: ^68^ Ga-DOTATATE PET/CT. AJR Am J Roentgenol. 2018;211(2):267-277. doi: 10.2214/AJR.18.19881 29975116

[6] Mettler FA Jr, Guiberteau MJ. Essentials of Nuclear Medicine and Molecular Imaging. 7th ed. Elsevier; 2019.

[7] Gusman M, Aminsharifi JA, Peacock JG, Anderson SB, Clemenshaw MN, Banks KP. Review of ^18^ F-fluciclovine PET for detection of recurrent prostate cancer. Radiographics. 2019;39(3):822-841. doi: 10.1148/rg.2019180139 31059396

